# Reviewed article: Alves NS, Silva EN, Melo GBT, Paulino MAS, Tuesta AA. Agenda for COVID-19 and long COVID research priorities in Brazil: results of wide consultation and Delphi consensus in 2022 and 2023. Epidemiol Serv Saude. 2025:34;e20240623

**DOI:** 10.1590/S2237-96222025v34e20240623.a

**Published:** 2025-06-20

**Authors:** Joao Carlos Alchieri

**Affiliations:** UFRN, Psicologia

**Table:** 

Questionnaire	Yes	No	Not applicable
Does the manuscript contain new and significant information to justify publication?	✓		
Does the Abstract (Summary) clearly and accurately describe the content of the article?	✓		
Is the problem significant and concisely stated?	✓		
Are the methods described comprehensively?	✓		
Are the interpretations and conclusions justified by the results?	✓		
Is adequate reference made to other work in the field?	✓		

**Table:** 

Rating	Excellent	Good	Average	Below average	Poor
Interest	✓				
Quality	✓				
Originality	✓				
Overall	✓				

## Manuscript Structure

Length of article is: Adequate

Number of tables is: Adequate

Number of figures is: Adequate

Please state any conflict(s) of interest that you have in relation to the review of this paper (state “none” if this is not applicable): 

O manuscrito apresentado caracteriza-se na objetividade dos processos investigativos quanto as definições de linhas de investigação na área de COVID-19, nos aspectos atuais de prevenção e de desenvolvimento tem investigações sobre consequências, riscos e as fragilidades no atendimento em saúde. Os autores empregam referências atuais diversificadas e pertinentes a temática em questão, brindando o leitor com informações ei distintas modalidades. A linguagem é objetiva e clara, com fluidez e concatenação de Ideais de modo a manter o leitor no contexto e foco das descrições.

Os aspectos metodológicos são pertinentes e atuais seguindo recomendações de consensos nacionais e internacionais na área que atendem perfeitamente ao objetivo planificado da investigação. Os aspectos referentes a participantes, cooptação, representação, critérios de inclusão e exclusão bem como os procedimentos éticos em pesquisa são apresentados adequadamente por parte dos autores.

Os resultados oriundos da investigação estão descritos de maneira adequada e representados graficamente por meio de tabelas que é seguro a compreensão por parte do leitor, tanto das etapas da investigação quanto dos resultados observados nas análises referentes às temáticas. O leitor se sente privilegiado em poder acompanhar o sequenciamento exposição de ideias deforma completa sem necessidade de revisão ou mesmo busca das informações em outros locais.

A discussão dos resultados apresentada é pertinente aos dados observados e compatível ao processo teórico fundamentado nas referências, tais características atendendo ao objetivo inicial e quanto as principais áreas e temáticas observadas por consenso na investigação. É possível compreender a necessidade e importância de linhas e áreas de investigação bem como a clareza com que definições a posteriori poderão ser tomadas diante destas informações.

A conclusão adequadamente apresentada tenta de forma sucinta contemplar uma quantidade considerável de dados e resultados observados. Trata-se de um manuscrito que com certeza embasará ulteriores investigações especialmente relacionadas as consequências do COVID-19.

## Comments

Um excelente estudo! 

